# Radiotherapy-induced uterine cacinosarcoma: A case report and review of the literature

**DOI:** 10.1016/j.ijscr.2022.106977

**Published:** 2022-04-01

**Authors:** Touimi Benjelloun Ahmed, Watik Fedoua, Asmaa Fouad, Oumaima Wajih, Houssine Boufettal, Sakher Mahdaoui, Naima Samouh

**Affiliations:** Gynecology Department, University Hospital Ibn Rochd, Faculty of Medicine and Pharmacy, Hassan II University of Casablanca, Morocco

**Keywords:** Radiation-induced uterine carcinosarcoma, Pelvic radiotherapy

## Abstract

Radiation therapy is a very effective treatment modality for cervical cancer, but unfortunately, ionizing radiation is associated with many side effects, including secondary cancer formation. We report a case of carcinosarcoma of the uterus in a woman with a history of pelvic irradiation for cervical carcinoma. A review of the literature was performed to present the incidence, optimal management, and prognosis for post-radiation uterine carcinosarcoma.

## Highlights

• Secondary neoplasm due to previous radiation therapy has been reported sporadically
• Endometrial tissue can persist after radiation therapy for cervical cancer and undergo neoplastic transformation
• Carcinosarcoma one of the least common but most serious consequences of radiotherapy for cervical cancer
• Carcinosarcoma can be avoided in the event of primary surgery or in the event of associated surgery

## 1 Introduction

Carcinosarcoma (CS) or mixed mullerian malignancy is a very rare and extremely aggressive tumor of the uterine body previously considered sarcomas, but now recognized as malignant tumors composed of metaplastic transformation of epithelial elements according to Leigh A. Cantrell [1]. It is an undifferentiated carcinoma that includes both carcinomatous and sarcomatous elements from a single malignant epithelial clone. It is a tumor of postmenopausal women, often discovered after postmenopausal metrorrhagia. The clinical presentation of uterine carcinosarcoma is not specific and imaging and pathological studies play an important role in the diagnosis. Its treatment is essentially surgical. There is currently no consensus on adjuvant therapy in its management. The prognosis is often poor, with 30–40% of cases having ectopic involvement at first presentation. Previous pelvic radiation has been identified as a risk factor for the development of development of uterine cacinosarcoma. A series of 23 patients who developed uterine cancer following pelvic radiation therapy has been reported; 35% of them had uterine carcinosarcoma compared to a baseline rate of 6% in the authors' population [3]. We present a rare case of radiation-induced uterine carcinosarcoma, the clinical, radiological and histological features of which we will study. In the light of this article, we wish to emphasize the importance of surgery in the treatment of cervical cancer and establish a relationship between pelvic radiotherapy and uterine carcinosarcoma.All our work has been reported in line with the SCARE criteria and guidelines [14].

## 2 Case report

**Fig. 1:**
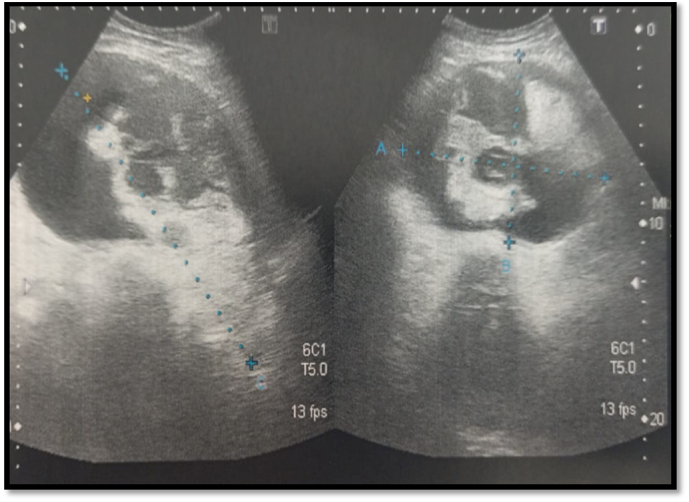
Abdomino-pelvic ultra-sound showing endocavitary intra uterine formation with a solid cystic component measuring 109 × 110 × 107 mm.

**Fig. 2:**
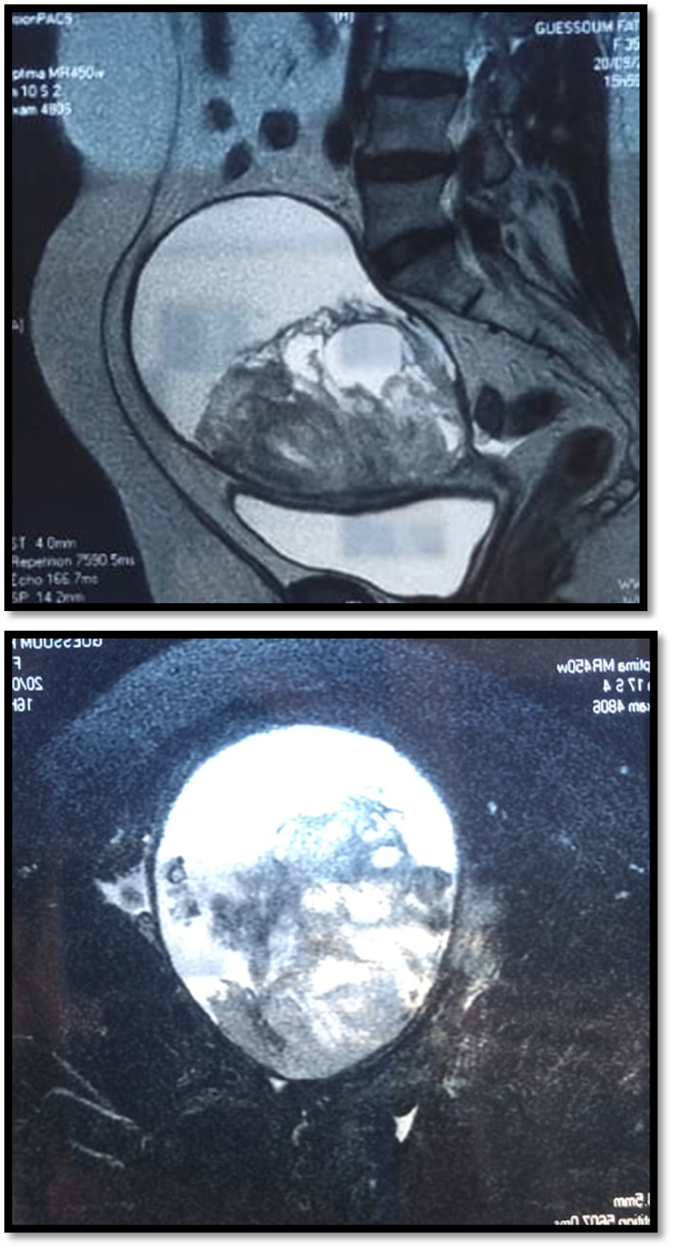
Abdomino-pelvic MRI.

G.F, 52 years old, mother of 3 children with a history of ulcerated squamous cell carcinoma of the uterine cervix, initially classified as stage II B according to the FIGO classification, treated exclusively with radiotherapy (66 GY) 10 years ago (no documents). The patient was admitted to our hospital for a pelvic mass discovered incidentally 5 months before her admission, without digestive, urinary or other associated gynecological signs. The whole evolving in a context of conservation of the general state. On clinical examination, an enlarged uterus was found 2 fingerbreadths below the umbilicus and a 6 cm prolapsed mass in the Cul de sac of Douglas and an aspirated cervix without bleeding on speculum examination. An abdomino-pelvic ultrasound (Fig. 1) was performed which showed a large solid-cystic mass superiorly and posteriorly on the left side measuring 12x9cm with regular contours, the solid part of which was of heterogeneous echostructure, non-vascularized on color Doppler without effusion in the douglas associated with minimal bilateral pyelo-caliceal dilatation on a normal looking kidney. An abdomino-pelvic magnetic resonance imaging (Fig. 2) was performed and found an enlarged uterus measuring 162 × 110 × 107 mm with a laminated aspect of the myometrium and fluid retention.

**Fig. 3:**
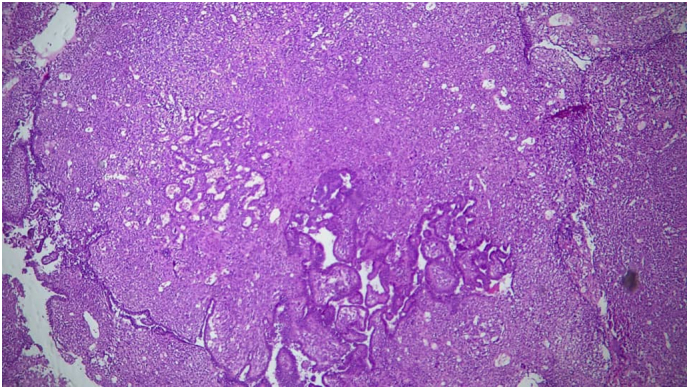
Mixed Mullerian tumor. Magnification ×100.

**Fig. 4:**
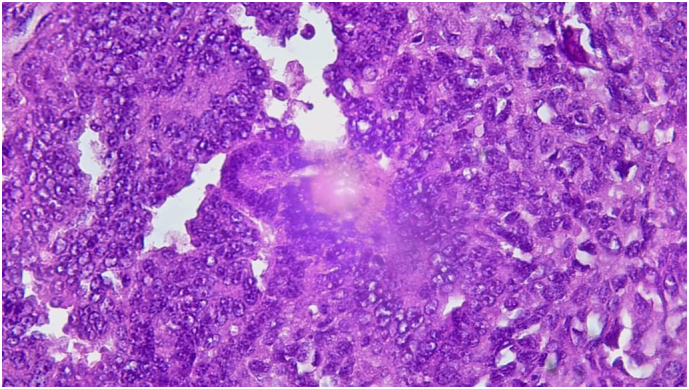
Mixed Mullerian tumor. Magnification ×200.

Presence of an endocavitary intrauterine formation with a solid cystic component measuring 109 × 110 × 107 mm, its tissue component is heterogeneous in T1 iso signal, T2 hyper signal, discretely enhanced after gadolinium injection, with an associated hemorrhagic component in T1 and T2 hypersignal. An endometrial biopsy was performed. It came back in favor of a poorly differentiated malignant tumor proliferation differentiated malignant proliferation suggestive of a mixed infiltrating (carinosarcoma) and partially necrotic mullerian tumor (Fig. 3, Fig. 4).

**Fig. 5:**
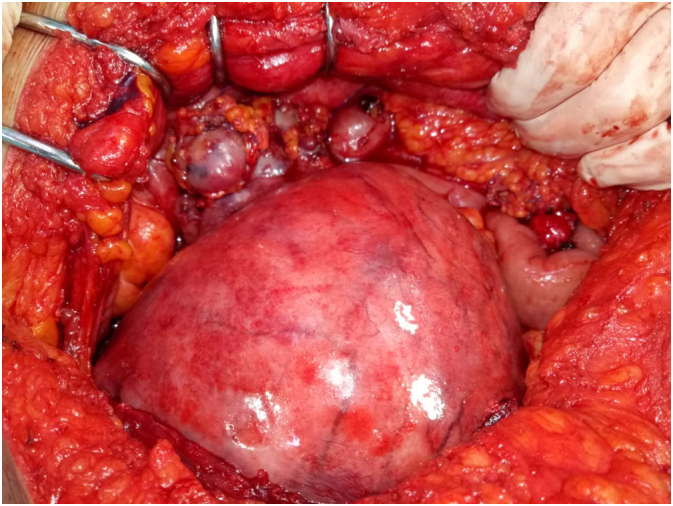
Surgical view of the uterus.

Hence the indication for surgical treatment which consisted of total hysterectomy without adnexal preservation with lymph node curage. Intraoperatively, the exploration (Fig. 5) was difficult because of the adhesions with aspect of carcinosis with a mass on the right ovary of necrotic aspect with tumor implants on the epiploon, posterior face of the bladder, anterior douglas cul de sac of the uterus extended to the parametrium, therefore multiple biopsies were performed and the postoperative follow-up was without particularity. Postoperative follow-up was unremarkable. At the final anatomopathology examination, it came back in favor of a poorly differentiated malignant tumor proliferation evoking a mixed infiltrating mullerian tumor (carcinosarcoma). The patient was classified according to the FIGO stage III A classification and then staffed to the multidisciplinary staff for radiochemotherapy.

## 3 Discussion

Uterine cervical cancer is one of the most common neoplasms in women that require irradiation to the whole pelvis even after complete cytoreduction. Secondary neoplasm due to previous radiation therapy has been reported sporadically in the literature since the 1980s [2], [6]. MMMT of the female reproductive system often represents a highly aggressive neoplasm that is characterized by a mixture of malignant epithelial and stromal elements comprising carcinomatous and sarcomatous neoplastic cells; the endometrium is usually the primary site. Nevertheless, endometrial tissue can persist after radiation therapy for cervical cancer and undergo neoplastic transformation. Hoffman et al. demonstrated that most post-radiation sarcomas were carcinosarcomas [15]. The carcinogenic effect caused by irradiation has been a subject of controversy and debate. Lorigan et al. suggested that for radiation to induce malignant change, the injury to individual cells must be sufficient to cause genetic mutation but insufficient to cause cell death; a situation that apparently arises at the margins of the radiation field. Boice et al. studied the risk of secondary malignancies as a consequence of radiation treatment of cervical cancer and reported that very high dose increased the risk of cancer of the bladder, rectum, vagina, uterine corpus, and bone. Moreover, according to Strom's study, the relative risk increased with time in organs close to and at an intermediate distance from the cervix, reaching a maximum after ≥30 years of follow-up since treatment. Most histologic types of secondary malignant tumors tend to have a long latent period and appear late (≥10 years after radiotherapy), except for radiation-induced leukemia. Yu reported that 10 cases of secondary MMMT occurred within 5–19 years after radiotherapy for cervical cancer [16]. Based on Hagiwara et al.'s study, the average latent period from initial irradiation to development of endometrial carcinoma is 13.4 years [17]. Naturally, the risk of secondary cancer associated with radiation peaks among long-term survivors and women irradiated at a relatively younger age. Nevertheless, patients treated by radiotherapy for cervical cancer are usually young and often survive for many years. However, as for post-irradiated uterine malignancies, delay in diagnosis may occur due to synechiae or stenosis of the cervix and cervical canal induced by previous irradiation, which prevent early onset of symptoms and require more effort to diagnose [7]. Although secondary malignancies induced by irradiation usually appear late, at >10 years after treatment, there is the possibility that it will occur earlier than expected. In this patient, it took only 5 years for the malignancy to arise [9]. The time interval is relatively shorter. For patients surviving cervical cancer, follow-up with regular Papanicolaou smear is the standard management. For those who received irradiation, not only longterm follow-up but also extreme caution is mandatory. For patients who present with any types of symptoms, aggressive and immediate investigation, including sonography and even tissue biopsy, is suggested in order to detect the possible occult malignancy as early as possible.

## 4 Conclusion

Carcinosarcoma one of the least common but most serious consequences of radiotherapy for cervical cancer, which can be avoided in the event of primary surgery or in the event of associated surgery in our case whose incidence is increasing in young women, raising concerns about the long-term consequences of its management.

## Provenance and peer review

Not commissioned, externally peer-reviewed.

## Sources of funding

None.

## Ethical approval

I declare on my honor that the ethical approval has been exempted by my establishment.

## Consent

Written informed consent for publication of their clinical details and/or clinical images was obtained from the patient.

## Author contribution

TOUIMI BENJELLOUN Ahmed: writing the paper

WATIK Fedoua: Corresponding author writing the paper

WAJIH Oumaima: writing the paper

BOUSADA Zakaria: writing the paper

BOUFETTAL Houssin: correction of the paper

MAHDAOUI Sakher: correction of the paper

SAMOUH NAIMA: correction of the paper

## Registration of research studies

researchregistry2464.

## Guarantor

DR FEDOUA WATIK.

## Declaration of competing interest

The authors declare having no conflicts of interest for this article.
